# Editorial: Cell size regulation: molecular mechanisms and physiological importance

**DOI:** 10.3389/fcell.2023.1219294

**Published:** 2023-05-18

**Authors:** Jette Lengefeld, Evgeny Zatulovskiy

**Affiliations:** ^1^ Helsinki Institute of Life Science, HiLIFE, Institute of Biotechnology, Faculty of Biological and Environmental Sciences, University of Helsinki, Helsinki, Finland; ^2^ Department of Medicine Huddinge, Center for Hematology and Regenerative Medicine, Karolinska Institutet, Stockholm, Sweden; ^3^ Department of Biology, Stanford University, Stanford, CA, United States

**Keywords:** cell size, aging, cell cycle, cell growth, mTOR

Cell size is an evident and universal property of all cells in nature, yet we still know surprisingly little about the molecular mechanisms controlling cell size. Even less is known about how changes in cell size affect cell physiology. Over the past decades, many studies have described that various genetic and chemical manipulations also alter cell size, but the significance of these size changes has often been overlooked when interpreting experimental results. Thus, we know very little of how cell size affects cellular processes. Now, it crystallizes that cell size is an indispensable cellular feature that globally affects cell physiology. In this Research Topic, we are presenting a collection of papers that provide an overview of the existing knowledge and new studies demonstrating how cell size is regulated and why cell size regulation is crucial for cell and tissue function (Figure 1).

**FIGURE 1:**
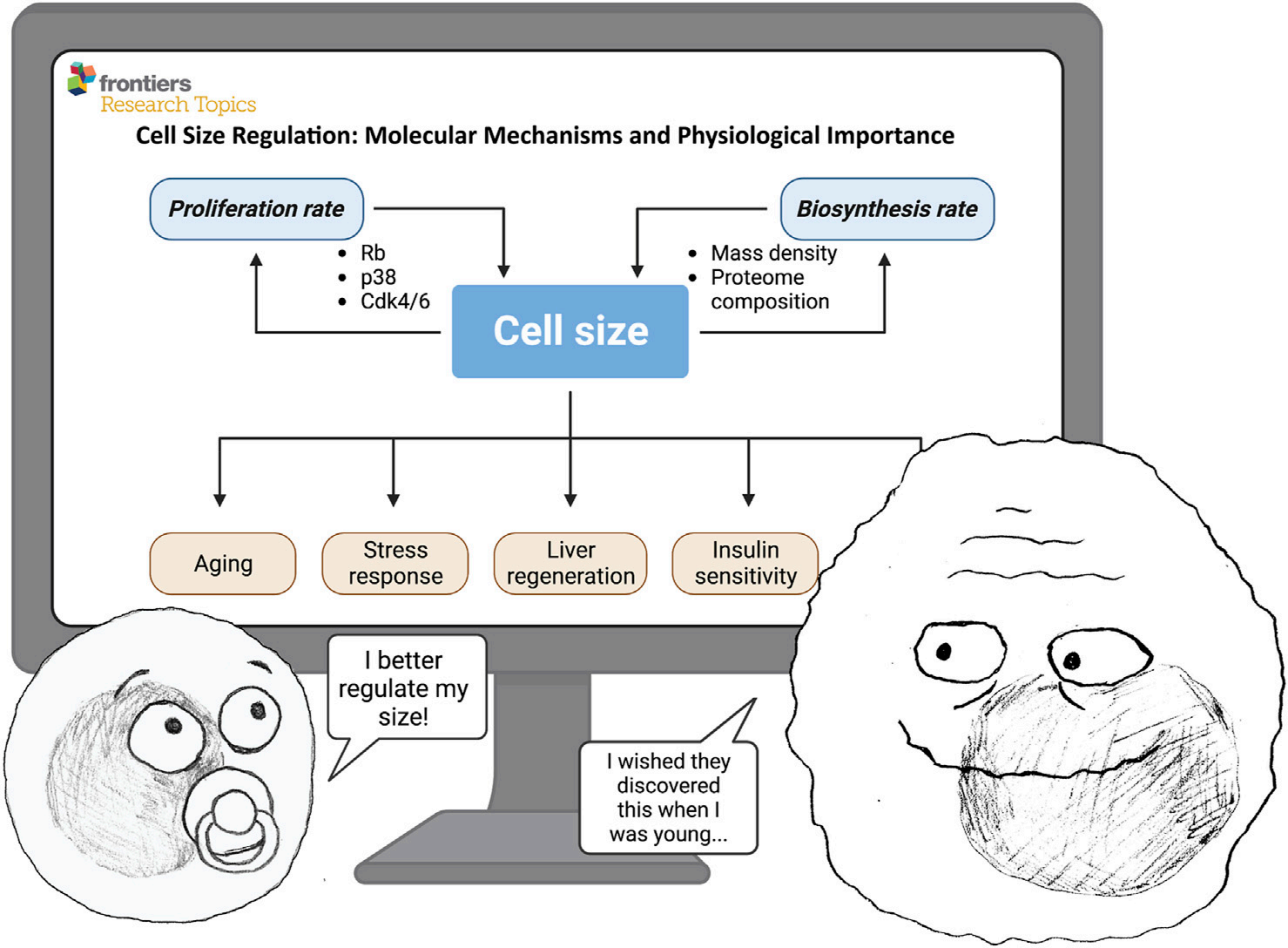
Cell size is determined by the balance between biosynthesis (cell mass accumulation rate) and proliferation (cell division rate). In turn, cell size can affect many important physiological processes—such as proliferation, growth rate, aging, stress response, regeneration, insulin sensitivity, and others. ©BioRender—biorender.com was used for image.

## How is cell size controlled?

Although cell size homeostasis has been studied for over a century, only with modern techniques are we able to gain more detailed and mechanistic insights into how cell size is regulated. The review by S. Liu et al. summarizes the historical findings and our current understanding of the molecular principles controlling cell size. An analogy of a ‘room thermostat’ is used to explain the two distinct modules of cell size regulation: one module sets the target average size for cells in a population, and another module senses cell size to ensure that cells do not deviate too far from the set size. Different types of mutations in size-related signaling pathways can either shift the mean size of the cells in the population or increase the size variation without changing the mean size (Liu et al., 2018; Tan et al., 2021). The review points out that a more comprehensive mechanistic understanding of what pathways sense and regulate cell size in a wild-type context still needs to be achieved in the future.

In global terms, cell size can be regulated in two possible ways: the cell can either adjust its biosynthesis rate (size-dependent growth) or its division rate (size-dependent cell cycle progression) (Cadart et al., 2018; Ginzberg et al., 2018; Zatulovskiy and Skotheim, 2020). To mechanistically understand how size affects cell cycle progression rates *in vivo*, the report by Zhang et al. followed up on the recent finding that size-dependent cell cycle progression in cultured cells is controlled by the dilution of a key cell cycle inhibitor - the retinoblastoma protein (RB) (Zatulovskiy et al., 2020). The authors demonstrate that RB dilution also plays an important role in controlling the size of mouse liver cells *in vivo*. This study confirms that growth-dependent dilution of cell cycle inhibitors is an evolutionary conserved mechanism that plays an important role in cell size regulation in different types of organisms (Schmoller et al., 2015; D’Ario et al., 2021).

The review by Fumagalli and Pende addresses the other branch of cell size control - the growth rate regulation. Specifically, the authors focus on one of the central nodes of mTOR signaling–the S6 kinase 1 (S6K1). They review proposed mechanisms underlying the effects of S6K1 on cell size and discuss the roles of S6K1 in connecting cell size with regeneration and aging. Evidence for a molecular program coordinating nucleic acid and protein synthesis, fat mass accumulation, retrograde control of insulin action, senescence program and cytoskeleton organization is revealed through the analysis of S6K1 targets.

While cell size is typically characterized either by the dry mass or by the volume of the cell these two parameters are regulated by different mechanisms and operate at different time scales (Cadart et al., 2019). The study by X. Liu et al. focuses on the biomass density dynamics in mammalian cells undergoing cell mass and cell volume changes. Using a novel technique that they developed and optimized - Normalized Raman Imaging (NoRI) - they demonstrate that besides the overall cell volume and mass, cells also very precisely control the intracellular mass density, which remains constant across many perturbations of cell biosynthesis and proliferation rates. Furthermore, they also observe that cellular senescence and starvation can alter the cell mass density, in agreement with previous reports (Neurohr et al., 2019; Neurohr and Amon, 2020; Oh et al., 2022). Thus, a stringent control of cell mass density is likely important for cell physiology.

## Why is cell size regulation important for cell function?

Cell size is tightly regulated within tissues (Xie and Skotheim, 2020), indicating that cell size control is crucial for cellular function. Yet, we know very little about how cell size affects cellular physiology and how cell size deregulation contributes to cellular dysfunctions. Aging is a prime example of the connection between cell size alterations and cellular dysfunction. The review by Davies et al. takes a thorough look through the body of literature published over the past decades to provide an overview of the connections between cell size and aging-related diseases. First, the review discusses the implications of recent studies suggesting that cellular enlargement promotes aging (Demidenko and Blagosklonny, 2008; Neurohr et al., 2019; Lengefeld et al., 2021; Lanz et al., 2022), and then highlights connections between cell size and established aging factors. Then, the focus is placed on publications reporting a correlation between cell size and aging-related diseases, crystallizing the potential of cellular enlargement as a new hallmark of aging. In line with this paper, the above-mentioned review by S. Liu et al. also highlights the connections between cell size, stemness, and lifespan/aging. These two papers underscore the need for future research to understand the correlative or causative links between cellular enlargement and function. Such research could affect other research areas that have so far overlooked the importance of cell size.

A related study by Zatulovskiy et al. sheds light on the mechanisms by which large cell size is connected to senescence and global proteome rearrangements. It was long assumed that senescent cells are large because they permanently exit the cell division cycle while continuing to grow in mass (Hernandez-Segura et al., 2018). However, using a quantitative proteomics approach, the authors demonstrated that the reversed causality also takes place–*i.e.*, large cell size itself leads to global proteome rearrangements that promote senescence (Lanz et al., 2022). Here, the authors show that an increase in cell size slows down cell growth, and this slowdown mediates many of the observed size-dependent changes in the cell proteome, including the senescence-related changes. These effects can be avoided if cell size increase is compensated by a corresponding increase in cell ploidy. These findings provide a possible rationale for why cells control their size: the further a cell gets from its size optimum, the more disbalanced its proteome becomes, which impairs cell fitness and function. For the same reason, cells that need to be large to perform their functions may become polyploid to avoid senescence and other negative consequences.

The slowdown of cell growth in large mammalian cells is accompanied by a selective reduction in the ribosome fraction. Terhorst et al. here demonstrate that selective downregulation of ribosomes also accompanies growth attenuation in cell-cycle-arrested budding yeast cells. Ribosome downregulation is orchestrated by a transcriptional program known as the Environmental Stress Response (ESR), which is activated in a size dependent manner. Interestingly, preventing downregulation of ribosome biogenesis did not rescue growth rates in large cells, suggesting that ribosomes are not rate limiting for protein synthesis in oversized cells.

A challenge in the cell size field is to understand how cell size affects the function of different organs *in vivo*. Two papers in this Research Topic approach this question by looking at cell size in the adipose tissue and in regenerating liver. The study by Lian et al. shows that liver regeneration after partial hepatectomy is impaired in fibrotic mouse livers. This reduction in tissue regeneration is accompanied by an increase in hepatocyte size and deregulated autophagy. However, the mechanistic and causal relationship between the fibrotic changes, cell size, autophagy, and regeneration remain to be elucidated.

The paper by Fryklund et al. demonstrates that diet-induced obesity in mice leads to inguinal adipocytes hypertrophy (increase in size), accompanied by an increased actin filamentation and reduced insulin-stimulated glucose uptake. Interestingly, when the animals were switched from 4 or 8 weeks of high-fat diet back to regular chow, those changes in cell size and insulin sensitivity reversed back to normal. However, if the animals were kept on a high-fat diet for 12 weeks, those changes could not be reversed anymore. Together with earlier observations that changes in pancreatic beta-cell sizes lead to reduced insulin production (Salans et al., 1968; Giordano et al., 1993), these findings point towards a possible contribution of cell size dysregulation to obesity-related diabetes.

## Summary

Overall, the papers presented in this Research Topic demonstrate how latest technological developments–such as Raman spectroscopy (X. Liu et al.), quantitative proteomics (Zatulovskiy et al.; Terhorst et al.), chemical and genetic screens (S. Liu et al. *;* Fumagalli and Pende) - provide an excellent opportunity for moving forward from phenomenological towards mechanistic studies of cell size. Our progress in understanding the functional roles of cell size regulation in tissues will strongly depend on our ability to adopt tractable and analyzable *in vivo* models for cell size research (Davies et al. *;* Lian et al. *;* Fryklund et al.; Zhang et al.).

### In memory of Angelika Amon - A brilliant researcher and role model

We would like to remember Angelika Amon, who died on the 29th of October 2020. Her pioneering work in the fields of cell cycle and cell size regulation was truly groundbreaking and she inspired countless individuals with her unwavering curiosity, perseverance, and passion for discovery. Angelika was also a devoted mentor and a fearless advocate for the rights of women and minorities in science. Her impact on our community and research will last a long time. We will remember her as a true pioneer and an inspiration to us all.
